# Chinese patent medicines for the treatment of the common cold: a systematic review of randomized clinical trials

**DOI:** 10.1186/1472-6882-14-273

**Published:** 2014-07-30

**Authors:** Wei Chen, Bo Liu, Li-qiong Wang, Jun Ren, Jian-ping Liu

**Affiliations:** Centre For Evidence-Based Chinese Medicine, Beijing University of Chinese Medicine, Beijing, China; Medical Care Center, Beijing Friendship Hospital, Capital Medical University, Beijing, China

**Keywords:** Common cold, Traditional Chinese medicine, Chinese patent medicine, Randomized controlled trial, Systematic review

## Abstract

**Background:**

Many Chinese patent medicines (CPMs) have been authorized by the Chinese State of Food and Drug Administration for the treatment of the common cold. A number of clinical trials have been conducted and published. However, there is no systematic review or meta-analysis on their efficacy and safety for the common cold to justify their clinical use.

**Methods:**

We searched CENTRAL, MEDLINE, EMBASE, SinoMed, CNKI, VIP, China Important Conference Papers Database, China Dissertation Database, and online clinical trial registry websites for published and unpublished randomized clinical trials (RCTs) of CPMs for the common cold till 31 March 2013. Revman 5.2 software was used for data analysis with effect estimate presented as relative risk (RR) and mean difference (MD) with a 95% confidence interval (CI).

**Results:**

A total of five RCTs were identified. All of the RCTs were of high risk of bias with flawed study design and poor methodological quality. All RCTs included children aged between 6 months to 14 years. Results of individual trials showed that Shuanghuanglian oral liquid (RR 4.00; 95% CI: 2.26 to 7.08), and Xiaoer Resuqing oral liquid (RR 1.43; 95% CI: 1.15 to 1.77) had higher cure rates compared with antivirus drugs. Most of the trials did not report adverse events, and the safety of CPMs was still uncertain.

**Conclusions:**

Some CPMs showed a potential positive effect for the common cold on cure rate. However, due to the poor methodology quality and the defects in the clinical design of the included RCTs, such as the lack of placebo controlled trials, the inappropriate comparison intervention and outcome measurement, the confirmative conclusions on the beneficial effect of CPMs for the common cold could not be drawn.

## Background

The common cold is one of the most widespread illnesses, and is most caused by rhinoviruses [1]. On average, adults may have two to four annually [2], and children may have six to ten colds a year [3]. Symptoms of the common cold include runny nose, cough, sore throat, fever and congestion.

Until now there is no proven treatment for the common cold. Previous studies did not show evidence of beneficial effect from antibiotics for the common cold, and prescription of antibiotics for the common cold may cause significant adverse effects [4, 5]. While some alternative treatments were used for the common cold, there was insufficient scientific evidence to support their use [3]. Simple analgesics and antipyretics are used for symptom control, such as ibuprofen [6] and acetaminophen/paracetamol [7].

Traditional Chinese medicine (TCM) has a long history of treating the common cold which can be traced back to more than 2000 years ago, and many Chinese herbs are believed to be effective. There are different categories of ‘cold’ in TCM, for example, 'wind-cold type cold', 'wind-heat type cold', and 'summer-heat and dampness type cold'. The categories are based on the cause and symptoms of cold, and the medications are different for different type of cold. Chinese patent medicine (CPM) is an important component of Chinese herbs, and is widely accepted by the Chinese people due to the convenience of application. More than 300 CPMs have been approved by the State Food and Drug Administration in China and dozens are listed in the ‘China national essential drug list’. It is an authorized list approved by the Ministry of Public Health of China, and provides the basis for the medicine used in medical institutions in China.

Until now, a number of clinical trials of CPM for the common cold have been conducted and reported. The aim of this systematic review is to provide evidence on potential benefits and harms of CPM for the common cold.

## Methods

### Search strategy and study selection

We searched CENTRAL (2012, Issue 12), MEDLINE, EMBASE, SinoMed, Chinese National Knowledge Infrastructure (CNKI), Chinese VIP information (VIP), China Important Conference Papers Database, and China Dissertation Database from their inception to 31 March 2013. We also searched websites of Chinese clinical trial registry (http://www.chictr.org/) and international clinical trial registry by U.S. National Institutes of Health (http://clinicaltrials.gov/) for ongoing registered clinical trials. The following search terms were used individually or combined: ‘cold’, ‘nasopharyngitis’, ‘acute viral rhinopharyngitis’, ‘acute coryza’, ‘shang feng (cold in Chinese)’, ‘wai gan (cold in Chinese)’, and ‘feng han (cold in Chinese)’, ‘Chinese Traditional’, ‘Chinese patent medicine’, ‘Oriental Traditional’, ‘herb’, ‘herbal medicine’, ‘clinical trial’, and ‘randomized controlled trial’. The search strategy of CNKI was listed in Appendix 1.

To ensure accuracy, two authors conducted the literature searching, study selection and data extraction independently, and the final results were double checked. Inconsistencies were resolved by negotiation or seeking third-party settlement. We extracted the following data: authors, title of study, year of publication, study size, age and sex of the participants, details of methodological information, name and component of Chinese patent medicines, treatment process, details of the control interventions, outcomes, and adverse effects.

### Inclusion criteria

Parallel randomized controlled trials (RCTs) of CPMs for the treatment of the common cold were included. There was no limitation on age or sex. Colds caused by influenza, acute bronchitis developing from a case of the common cold, and upper respiratory tract infection caused by bacteria were excluded. We restricted CPMs as those listed in ‘China national essential drug list 2012’. CPMs is defined as those mainly produced by modern manufacturing methods. These are produced as different preparations of herbal medicines such as powder, granule, pastille, tablet, and capsule.

The comparison interventions included no treatment, placebo or conventional medication. Combined therapy of CPMs and other interventions compared with other interventions was also included. The outcome measures were fever clearance time (the time between commencing treatment and temperature returning to normal), cure rate, time to resolution of individual symptoms (cough, nasal congestion, nasal drainage and sore throat) and adverse events. Publications that report same data were regarded as multiple publications and were excluded. There was no restriction on language and publication type.

### Trial quality assessment

Two authors evaluated the quality of included trials independently. We used the Cochrane 'Risk of bias' tool for assessing bias with consideration of the following aspects: random sequence generation, allocation concealment, blinding of participants and personnel, blinding of outcome assessment, incomplete outcome data, and selective reporting [8]. The quality of all the included trials was evaluated as to be low/unclear/high risk of bias according to how these above criteria were met.

### Data analysis

We used the relative risk (RR) with 95% confidence intervals (CI) for dichotomous data, and mean difference (MD) with 95% CI for continuous outcomes. Revman 5.2 software was used for data analyses. For RCTs with good homogeneity on study design, participants, interventions, control, and outcome measures, meta-analysis was planned to be conducted. If at least ten trials were available for a meta-analysis, we would assess for the likelihood of publication bias by constructing funnel plots [8].

## Results

### Description of studies

**Figure 1:**
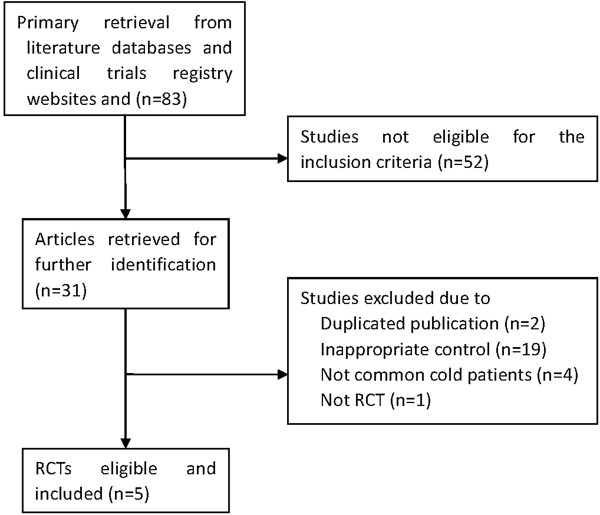
**Flow chart of included studies in this systematic review.**

A total of 33 CPMs were listed in ‘China national essential drug list 2012’ for the treatment of the common cold, and clinical studies of the 33 CPMs were searched from the above eight databases and two clinical trials registry websites. A flow chart depicts the search process and study selection (Figure 1). After primary searches, 83 citations were identified, and the majority was excluded due to obvious ineligibility from reading title/abstract. Full text papers of 31 studies were retrieved. At last, a total of 5 RCTs including 5 different CPMs [9–13] were included. These 5 CPMs were Chaihu injection [9], Ganmao Qingre granules [10], Shuanghuanglian oral liquid [11], Xiaoer Baotaikang granules [12], and Xiaoer Resuqing oral liquid [13]. One four-armed RCT [9] was included which compared Chaihu injection given by acupoint block, Chaihu injection given by intramuscular injection, saline given by acupoint block, and ribavirin given by intramuscular injection. The compositions and indications of the 5 CPMs included in the review were shown in Table 1. We collected the data of group that received Chaihu injection given by intramuscular injection and group that received ribavirin given by intramuscular injection. The search for ongoing registered trials identified no trial.

**Table 1:** **Compositions and indications of CPMs included in the review for the common cold**

Name of CPMs	Compositions	Indications
Chaihu Injection	*Radix Bupleuri*	For the colds, influenza, malaria and other fever.
Ganmao Qingre granules	*Schizonepeta tenuisfolia Briq*, *Herba Mentha haplocalyx*., *Radix Saposhnikoviae*, *Radix Bupleuri*, *Folium Perillae*, *Radix Pueraria lobata* (*Willd*) *Ohwi*., *Radix Platycodonis*, *Semen Armeniacae Amarum*, *Radix Angelica dahuricae*, *Herba corydalis Bungeanae*, *Rhizoma Phragmites communis Trin*	For the wind-cold cold, headache, fever, aversion to cold, body pain, runny nose, cough and dry throat.
Shuanghuanglian oral liquid	*Radix Scutellaria baicalensis Georgi*, *Flos Lonicerae*, *Fructus Forsythiae*	For the cold induced by external contraction wind-heat, such as fever, cough, pharyngalgia.
Xiaoer Baotaikang granules	*Fructus Forsythiae*, *Radix Rehmanniae*, *Bupleurum marginatum*, *Radix Scrophulariae*, *Folium Mori*, *Bulbus Fritillaria thunbergii Miq*., *Herba Taraxaci*, *Baphicacanthus cusia* (*Nees*) *Bremek*, *Onosma*, *Radix Platycodonis*, *Semen Raphani*, *Radix Glycyrrhizae*	For children with external contraction wind-heat, such as fever, nasal discharge, cough
Xiaoer Resuqing oral liquid	*Radix Bupleuri*, *Radix Scutellaria baicalensis Georgi*,*Radix Isatidis*, *Radix Pueraria lobata* (*Willd*) *Ohwi*., *Flos Lonicerae*, *Cornu Bubali*, *Fructus Forsythiae*, *Radix et Rhizoma Rhei*	For children with cold induced by external contraction wind-heat, such as fever, headache, throat swelling and pain, nasal congestion and nasal discharge, cough, dry stool.

The characteristics of included trials are listed in Table 2. A total of 633 participants with the common cold were involved with the average number of 127 per trial, ranging from 60 to 253. All RCTs included children aged between 6 months to 14 years. One RCT [9] did not provide the age of included children. All RCTs included outpatients. Three RCTs [9, 12, 13] diagnosed the common cold by criteria issued by the State Administration of Traditional Chinese Medicine of the People’s Republic of China, one RCT [10] diagnosed the common cold by criteria from textbook, and one RCT [11] diagnosed the common cold based on Clinical symptoms and laboratory test results. One trial included wind-cold type of common cold [10], one trial included wind-heat type of common cold [13], while the other three trials did not provide information on patients’ syndrome differentiation. The total treatment duration ranged from 3 to 5 days. The control interventions included antibiotics, antivirus drugs, and antipyretic and analgesic drugs. No placebo controlled trial was identified. The reported outcome measurements included fever clearance time, cure rate, and adverse events.

**Table 2:** **The characteristics of included RCTs**

Name of CPMs	Study ID	Diagnostic criteria	TCM pattern differentiation	Patients	Comparator	Outcomes	Adverse events
Chaihu injection (acupoint block in T1 or intramuscular injection in T2)	Lv [9]	Guiding principle of clinical research on new drugs of traditional Chinese Medicine*	Not reported	N = 253 (100 in T1, 52 in T2, 50 in C1, and 51 in C2); M/F: not reported; Age: not reported;	C1: saline acupoint block; C2: ribavirin	Fever clearance time and cure rate	Not mentioned.
Ganmao Qingre granules	Di [10]	Chinese clinical integrative medicine Textbook	Wind-cold type of common cold	N = 60 (30/30); M/F: 18/12 in T and 16/14 in C; Age: 6 months-12 years	Pediatric paracetamol	Cure rate	Not mentioned.
Shuanghuanglian oral liquid	Wang [11]	Clinical symptoms and laboratory test results	Not reported	N = 100 (50/50); M/F: not reported; Age: 6 months-14 years	Ribavirin injection	Cure rate	No adverse event was identified.
Xiaoer Baotaikang granules	Wu [12]	Criteria of diagnosis and therapeutic effect of TCM diseases*	Wind-heat type of common cold	N = 100 (50/50); M/F: not reported; Age: 4.28 ± 3.37 years in T, and 4.56 ± 3.44 years in C.	Amoxicillin granules	Cure rate	Not mentioned.
Xiaoer Resuqing oral liquid	Li [13]	Diagnostic criteria for high fever of external contraction (Waigan Gaore in TCM syndroms)*	Not reported	N = 120 (65/55); M/F: 75/45; Age: 8 months-7 years	Moroxydine tablets	Cure rate	Not mentioned.

*Issued by the State Administration of Traditional Chinese Medicine of the People’s Republic of China.

### Methodological quality

**Figure 2:**
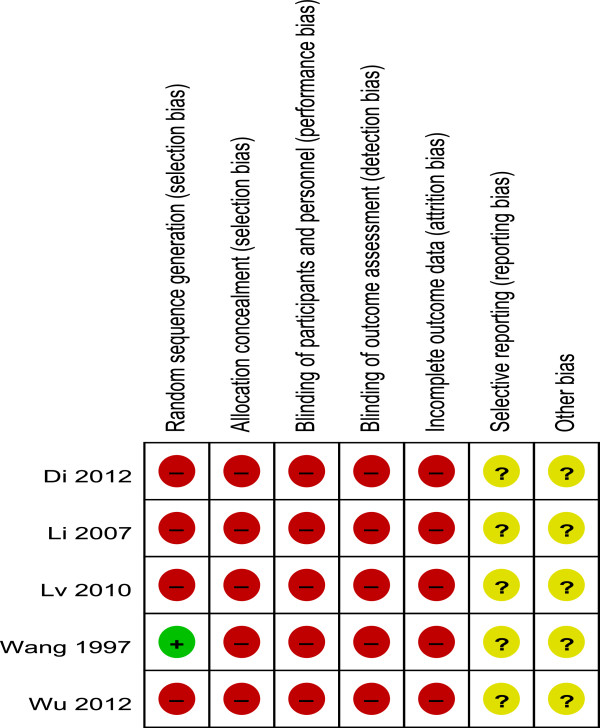
**Risk of bias summary about each risk of bias item for each included study.**

All of the included trials were assessed to be of generally poor methodological quality. All the RCTs mentioned that ‘participants were randomized into groups’, but did not report the detailed methods for sequence generation. We contacted the authors of the included RCTs, one trial [11] claimed that they drew lots to assign the participants; the other trials did not respond to our inquiry. Allocation concealment and blinding was not mentioned in any RCT. And because of the different formulation of CPMs and control intervention, it was not likely to conduct blinding either to investigator or to patients. No trial reported drop-out or withdrawals, or mentioned intention-to-treat analysis. Selective reporting was unclear because we could not access to the protocols of included RCT. All the included RCTs did not register in relevant authoritative websites prior to the start of the study. The risk of bias summary about each risk of bias item for each included study is shown in Figure 2.

### Effect estimates

1. 1 CPM vs. antivirus drugs

### Fever clearance time

One RCT [9] reported the effect of Chaihu injection given by intramuscular injection on defervescence. Results showed that there was no difference between Chaihu and ribavirin injection on fever clearance time (hours) (MD −0.99, 95% CI: −6.31 to 4.33).

### Cure rate

**Figure 3:**
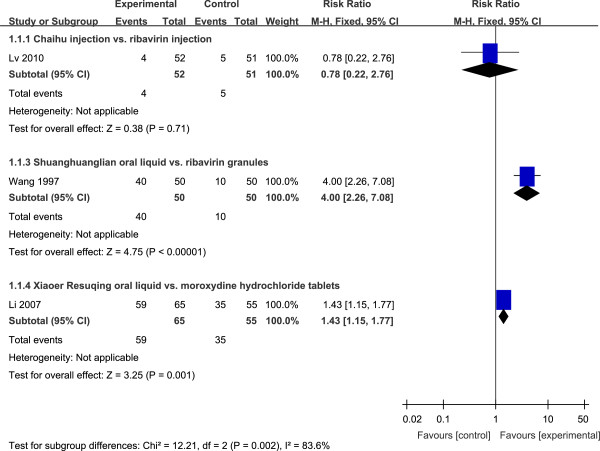
**Forest plot of comparison of CPM with antivirus drugs on cure rate.**

Three trials [9, 11, 13] compared CPMs with antivirus drugs on cure rate. Results showed that there was no difference between Chaihu injection and ribavirin injection (RR 0.78, 95% CI: 0.22 to 2.76), Shuanghuanglian oral liquid had higher cure rate compared with ribavirin injection (RR 4.00; 95% CI: 2.26 to 7.08), and Xiaoer Resuqing oral liquid had higher cure rate compared with moroxydine tablets (RR 1.43; 95% CI: 1.15 to 1.77) (Figure 3).

Meta-analysis was not performed due to the heterogeneity in experimental and control interventions among included trials.

2. CPM vs. antipyretic and analgesic drugs

### Cure rate

One trial [10] compared CPMs with antipyretic and analgesic on cure rate. Results showed that there was no difference between Ganmao Qingre granules and pediatric paracetamol on cure rate (RR 1.31; 95% CI: 0.78 to 2.19).

3. CPM vs. antibiotics

### Cure rate

One trial [12] compared CPMs with antibiotics on cure rate. Results showed that Xiaoer Baotaikang granules had higher cure rate compared with amoxicillin granules (RR 2.67; 95% CI: 1.56 to 4.55).

### Adverse events

One RCT [11] stated that no adverse event was identified. The remaining four RCTs did not mention whether they monitored the adverse events.

### Funnel plot

We did not conduct funnel plots due to the inadequate number of included trials.

## Discussion

In this review, we found out that only 5 out of 33 CPMs recommended in the ‘China national essential drug list’ for the common cold had scientific clinical evidence to support their use. However, due to the poor methodology quality and defects in the clinical design, such as lack of placebo control, the inappropriate comparison intervention and outcome measurement, the confirmative conclusions on the beneficial effect of CPMs for the common cold could not be drawn. In addition, all the included RCTs enrolled children aged between 6 months to 14 years. The effect of CPMs for adult patients could not be drawn from this review.

In our review, we searched all CPMs listed in the ‘China national essential drug list 2012’. The reason was that ‘China national essential drug list’ was the authorized list approved by the Ministry of Public Health of China and was provided as the basis for the medicine used in medical institutions in China. However, although 33 CPMs were recommended in the ‘China national essential drug list 2012’ for the common cold, only 5 were found have previous clinical evidence. Our results revealed the lack of evidence for clinical use and policy making of TCM in China.

We thought the possible reasons of the inconclusive therapeutic effect of the above CPMs for the common cold might be as follows: Firstly, all the included trials were assessed to be of poor methodology quality. No RCT stated randomization procedure except one [11]; the rest just mentioned that ‘the patients were randomized into two groups’ without providing detailed information to judge whether randomization was conducted properly. Allocation concealment and blinding was not mentioned in any RCT. And because of the different formulation of CPMs and control intervention, it is not likely to be possible to achieve blinding either to investigator or to patients. Therefore, both the investigator and the patients know exactly if they are taking CPMs or conventional medicines. In China, people generally have strong expectations of benefit from CPMs than conventional medicine due to the deeply rooted belief in TCM. We believed that these preferences and expectations might positively bias participants' later responses, especially when the outcome measurement was a more subjective index such as cure rate. Fever clearance time, in contrast, was an objective outcome and was less likely to be affected by participants’ expectations. However, only one trial reported the data and the result showed that there was no difference between Chaihu and ribavirin injection on fever clearance time. Previous studies had proven that methodologically poorly designed trials would demonstrate larger differences between experimental and control groups than those conducted rigorously [14, 15], therefore, we should use caution when interpreting the the small improvements in outcomes in our review.

Secondly, there was lack of placebo control in the included RCTs. The common cold is a mild and self-limiting disease and does not have the proven effective intervention. For this kind of disease, we think a placebo controlled trial is the best choice. In this review, no placebo controlled trial was identified, while the abuse of antibiotics and misuse of antiviral deserved our concern. In our review, the control interventions included antibiotics, antivirus drugs, and antipyretic and analgesic drugs. It is known that antibiotics have no effect against viral infections and thus have no effect against the viruses that cause the common cold. However, in China, antibiotics are quite commonly used for the common cold in clinical practice even if in most of the cases there is no sign of complications of bacterial infection. Some of the reasons that antibiotics are so commonly prescribed include people's expectations for them, physicians' desire to help, and the difficulty in excluding complications that may be amenable to antibiotics. In our review, one RCT [12] used amoxicillin granules as the control intervention, however, it did not report whether there were simultaneous bacterial infections. There are also no effective antiviral drugs for the common cold that have been proven by clinical trials [3]. In this review, two antiviral medicines (ribavirin and moroxydine) were used as the control intervention. In the UK and USA, ribavirin is only used for severe respiratory infections such as respiratory syncytial virus bronchiolitis in infants and children or severe acute respiratory syndrome (SARS) (or for chronic hepatitis) [16–18]. Moroxydine is originally developed as an influenza treatment. In our review, all the patients were enrolled as having simple the common cold, no data was provided on whether the patients was having complications or in high risk categories that needs to be treated by ribavirin and moroxydine, and from the average short treatment period (3 to 5 days), it was less likely that these treatments were for severe respiratory infections. It has been proved that antibiotics could cause significant adverse effects in adults when given for the common cold [5], and the adverse events of ribavirin was found in treating SARS [19]. Therefore, the risk-benefit of using drugs such as antibiotics and antiviral drugs deserves more attention for future studies of the common cold.

Thirdly, the outcome measurements of the included RCTs were ambiguous and susceptible to bias. All RCTs used ‘cure rate’ as the outcome measurement. It was a composite outcome including the clinical symptom disappearance and abatement of fever. It was not an internationally recognized outcome measurement but was commonly used in Chinese TCM trials. However, subtle differences existed in the criteria or cut point in different trials, which made it difficult to interpret the effects of CPMs. Moreover, this outcome measurement was susceptible to subjective bias when assessing the improvement of clinical symptoms. The included RCTs did not state that they have independent outcome assessors. We believed that the outcome assessors were the investigators themselves. This approach was very easy to introduce bias, especially for subjective outcome such as cure rate. Another thing worth noting in this review is that Chinese TCM researchers pay too much attention on the body temperature. Even for the composite outcome measurement of ‘cure rate’, body temperature was always a vital component, though different definitions were used. However, fever was just one of the symptoms of the common cold and might not be the primary one because the common cold caused a range of symptoms such as coughing, sore throat, runny nose and sneezing. Future TCM researchers should be encouraged to use well validated outcome measurements and to consider all the related symptoms, for example, patient-reported daily symptom diary including symptom variables.

Finally, the diagnostic criteria used in the included RCTs were quite diverse. All diagnostic criteria were based on clinical symptoms and laboratory test results. No trial used virological examination. Possibility existed that participants with other acute respiratory infections might have been misdiagnosed as having simple common cold and were recruited. In addition, the report of patients’ syndrome differentiation ('bianzheng' in TCM, a specific diagnosis in TCM) was inadequate. In the practice of TCM, herbal preparations should match the type of syndrome differentiation, and this was thought to be the advantages of TCM. Chinese medicine practitioners believed that treating patients without syndrome differentiation would impair the advantages of Chinese herbs. In our review, only two trials reported patients’ syndrome differentiation, the relationship between the efficacy and the pattern of the common cold could not be investigated due to the lack of a group with the opposite pattern of syndromes. In addition, no trial reported whether they had quality control for the herbal preparations or for the herbal products.

The reporting of adverse events was not adequate. Only one RCT [11] stated that no adverse event was identified. The remaining four RCTs did not mention whether they monitored the adverse events. Conclusions about the safety of CPMs cannot be drawn from this review.

In our review, all the included trials were conducted in China and published in Chinese, no English paper was identified, and no 'negative' study was included. We did not conduct funnel plots due to the inadequate number of included trials, however, possibility of publication bias could not be excluded considering the fact that all the trials claimed positive effect favoring CPMs though some of the trials turned out to be negative when analyzed by standard statistical techniques using risk ratios or mean differences.

We noticed that a systematic review that assessed the effect of Chinese medicinal herbs for the common cold has been published [20]. In this review, a total of 17 trials were included. However this review included both CPMs and individually prescribed herbal formulae. Most of them were not the recommended CPMs listed in the ‘China national essential drug list 2012’ that was the accepted reference point for the medicines used in medical institutions in China. The CPMs in this review did not include our CPMs. We thought the reason might be the difference in the search strategy and the date of searching. Most of our included RCT were conducted after 2010 which is beyond the searching date of this previous review.

In summary, we could not take the beneficial effect of CPMs for the common cold as a confirmative conclusion. For mild and self-limitating disease like the common cold, a placebo controlled trial is the best study design to determine the efficacy of intervention. In addition, future TCM researchers should place more emphasis to the selection of outcome measurement, control intervention, and report trials according to the CONSORT Statement [21].

## Conclusions

Some CPMs showed a potential positive effect for the common cold on cure rate. However, due to the poor methodology quality and the defects in the clinical design of the included RCTs, such as the lack of placebo controlled trials, the inappropriate comparison intervention and outcome measurement, the confirmative conclusions on the beneficial effect of CPMs for the common cold could not be drawn.

### Consent

Written informed consent was obtained from the patient for the publication of this report and any accompanying images.

## Appendix 1: CNKI search strategy

1. exp Randomized Controlled trials/all subheadings
2. Random*
3. 1 or 2
4. exp traditional Chinese medicine/all subheadings
5. exp Chinese patent medicine/all subheadings
6. exp herbal medicine/all subheadings
7. or/4-6
8. common cold
9. nasopharyngitis
10. acute viral rhinopharyngitis
11. acute coryza
12. shang feng
13. wai gan
14. feng han
15. or/8-14
16. Human
17. 3 and 7 and 15 and 16
